# Inconsistent condom use with casual partners among men who have sex with men in Brazil: a cross-sectional study

**DOI:** 10.1590/1980-549720230019

**Published:** 2023-03-24

**Authors:** Bruna Hentges, Daniela Riva Knauth, Alvaro Vigo, Luciana Barcellos Teixeira, Andréa Fachel Leal, Carl Kendall, Laio Magno, Inês Dourado, Ligia Kerr

**Affiliations:** Universidade Federal do Rio Grande do Sul, School of Medicine, Epidemiology Undergraduate Program – Porto Alegre (RS), Brazil; Universidade Federal do Rio Grande do Sul, Department of Social Medicine – Porto Alegre (RS), Brazil.; Universidade Federal do Rio Grande do Sul, Department of Statistics – Porto Alegre (RS), Brazil.; Universidade Federal do Rio Grande do Sul, Department of Public Health – Porto Alegre (RS), Brazil.; Universidade Federal do Rio Grande do Sul, Institute of Philosophy and Human Sciences. Porto Alegre (RS), Brazil.; Universidade do Ceará, School of Medicine, Department of Collective Health. Fortaleza (CE), Brazil.; Universidade do Estado da Bahia, Department of Life Sciences, Salvador (BA), Brazil.; Universidade Federal da Bahia, Health Collective Institute, Salvador (BA), Brazil.; Universidade Federal do Ceará, Department of Community Health, Fortaleza (CE), Brazil.

**Keywords:** Condoms, Sexual and gender minorities, HIV infections, Latin America, Preservativos, Minorias sexuais e de gênero, Infecções por HIV, América Latina

## Abstract

**Objective:**

This study aimed to evaluate factors associated with inconsistent condom use with casual partners in a population of men who have sex with men (MSM) in Brazil.

**Methods:**

In 2016, 4,176 MSM >18 years were enrolled in 12 capitals of Brazil using a Respondent Driven Sampling (RDS) method. For the construction of the outcome, we evaluated questions about condom use in all anal intercourse (receptive and insertive) in the previous six months and the last sexual intercourse. Estimates were calculated using a weighted complex sample design. We performed a logistic regression analysis to determine the associations between sociodemographic and behavioral factors and inconsistent condom use in sexual relationships with casual male partners.

**Results:**

More than half of our sample (50.8%) had not used condoms consistently with casual partners in the previous six months. Inconsistent condom use was significantly associated with: low education (weighted odds ratio — wOR: 1.55; 95% confidence interval — CI 0.99–2.40), lack of counseling on sexually transmitted infections STI (wOR: 1.51; 95%CI 1.05–2.17), non-use of condoms at sexual debut (wOR: 3.05; 95%CI 2.12–4.40) and moderate and high perceived risk for HIV (wOR: 1.51; 95%CI 1.07–2.14). Higher age was negatively associated with inconsistent condom use (wOR=0.97, 95%CI 0.89–0.99).

**Conclusion:**

Despite being an individual behavior, condom use is related to factors beyond the individual scope. HIV/Aids prevention policies should focus on younger MSM, providing qualified information about condom use, preferably before the beginning of their sexual life.

## RESUMO

**Objetivo:**

Este estudo tem como objetivo avaliar os fatores associados ao uso inconsistente do preservativo com parceiros casuais em uma população de homens que fazem sexo com homens (HSH) no Brasil.

**Métodos:**

Trata-se de um estudo transversal, com método de amostragem *Respondent Driven Sampling* (RDS), desenvolvido em 12 capitais do Brasil, com 4.176 HSH. Para a construção do desfecho, foram avaliadas questões sobre o uso do preservativo em todas as relações anais (receptivas e insertivas) nos últimos seis meses e a última relação sexual. As estimativas foram calculadas usando um desenho amostral complexo ponderado. Realizamos uma análise de regressão logística para determinar as associações entre fatores sociodemográficos e comportamentais e o uso inconsistente do preservativo.

**Resultados:**

Mais da metade da nossa amostra (50,8%) não usou preservativo de forma consistente com parceiros casuais nos últimos seis meses. O uso inconsistente do preservativo foi significativamente associado a: falta de aconselhamento sobre infecções sexualmente transmissíveis (*weighted odds ratio* — wOR: 1,51; intervalo de confiança — IC95% 1,05–2,17), não uso de preservativo na primeira relação sexual (wOR: 3,05; IC95% 2,12–4,40) e autopercepção de risco para o HIV como moderado e alto (wOR: 1,51; IC95% 1,07–2,14). Maior idade foi negativamente associada ao uso inconsistente do preservativo (wOR=0,97, IC95% 0,89–0,99).

**Conclusão:**

Apesar de ser um comportamento individual, o uso do preservativo está relacionado a fatores além do âmbito privado. As políticas de prevenção ao HIV/AIDS devem focar nos HSH mais jovens, fornecendo informações qualificadas sobre o uso do preservativo, preferencialmente antes do início da vida sexual.

## INTRODUCTION

Forty years after the first cases of HIV/Aids were recorded, the disease is still a challenge for health managers. Global HIV/Aids epidemic data show a downward trend in infection and mortality rates for the general population, with a 31% decrease in incidence between 2010 and 2021^1^. However, these data do not reflect disparities among other groups. In Latin America, for example, where the epidemic is concentrated in key populations (men who have sex with men — MSM, sex workers, drug users, transgender women, and people deprived of their liberty), there has been no reduction in the rates of new infections since 2010^1^.

MSM are a group particularly affected by HIV/Aids. This population itself represents 23% of new HIV/Aids infections globally, and 46% of new infections in Latin America, with a prevalence ranging from 1.2 to 32.6% in the countries of this region^2^. In Brazil, the prevalence of HIV/Aids among MSM is 18.4%, with a tendency to increase in recent years^3^, while the prevalence of the disease among adults in the general population remains stabilized at 0.6%^4^.

In the last two decades, biomedical innovations have contributed to increasing the available options for the prevention of HIV/Aids, such as sexual post-exposure prophylaxis (PEP), sexual pre-exposure prophylaxis (PrEP), and HIV self-testing. Even so, the use of the male condom in sexual relations is considered an accessible preventive method, efficiently reducing the sexual transmission of HIV and other sexually transmitted infections (STI)^5^. Especially in countries with fewer health resources, promoting condom use can be a critical HIV/Aids prevention mechanism^6^. When used correctly and consistently, the effectiveness of male condoms in anal intercourse is estimated to be 73 to 99.6% (95% confidence interval — CI 69–101)^7^. However, the literature points out that many MSM, including the young ones, do not use male condoms or use them inconsistently^8,9,10^. Inconsistent condom use with HIV-positive male partners or unknown serology does not provide protection^11^.

The relationship established with the sexual partner seems to be an essential component of condom use among MSM. Studies suggest that there is a higher use of condoms in sexual relations between casual partners when compared to steady partners, due to a possible perception of lower risk and monogamy between the parties^12,13^. However, few studies seek to understand the factors associated with inconsistent condom use among casual partners, especially in low- and middle-income countries^14,15^. This article aims to analyze the factors associated with inconsistent condom use with casual partners in a sample of MSM in Brazil to understand this phenomenon.

## METHODS

### Population and study design

The analyzed data come from a cross-sectional study entitled “National study of behaviors, attitudes, practices, and prevalence of HIV, Syphilis and Hepatitis B and C among men who have sex with men”^16^. The study was carried out in 12 capitals, covering the five regions of Brazil: Manaus, Belém, (North Region); Fortaleza, Recife, Salvador, (Northeast Region); Brasília, Campo Grande (Central-West region); Belo Horizonte, Rio de Janeiro and São Paulo (Southeast Region); and Curitiba and Porto Alegre (South Region). Data collection took place in 2016. The final sample of the study was 4,171 MSM.

Participants were recruited using the Respondent-Driven Sampling (RDS) methodology. The main requirement of RDS is that the target group members are connected through networks of social relationships^17^. To analyze this data, each participant needs to provide accurate information about the total number of people in this network to recruit for the study. Recruitment can be estimated for each participant, and weight calculated through this information.

To be eligible for participation, respondents had to be men aged 18 years or older, who reported having had oral or anal sex with another man in the 12 months prior to the survey, and resided, worked, or studied in one of the cities where the research was conducted. With the help of trained interviewers, each participant answered a questionnaire about sexual behavior, violence, knowledge about HIV/Aids, and drug and alcohol use. Details about the study methodology can be found in a previously published article^18^. The overall study was approved by the Committee on Research Ethics of the Federal University of Ceará, accredited by the National Commission on Research (#1.024.053(23/06/2015)). All respondents signed a consent form to participate in the research.

### Outcome and explanatory variables

The outcome of interest is inconsistent condom use with casual male partners. This variable was constructed by combining three variables: condom use in all receptive anal intercourse in the previous six months, condom use in all insertive anal intercourse in the previous six months, and condom use at the last sexual intercourse, regardless of the type of relationship. Use was considered inconsistent when the respondent answered “most of the time,” “sometimes,” “rarely,” or “never,” and consistent use when the answer was “always use condoms.” The inconsistent use variable was dichotomized into yes and no.

In the analysis, sociodemographic and behavioral variables were considered. The sociodemographic variables were: age; schooling, categorized as low (less than 12 years of schooling, corresponding to incomplete high school) or high (12 years or more of schooling, corresponding to complete high school or higher); socioeconomic class (Brazilian criteria as higher [A-B] or lower [C/D-E]); self-reported skin color (categorized as white and non-white); practicing any religion and relationship status (married or in a stable union; and single/divorced or separated). For the analysis of the entire sample, age was categorized into three groups, according to the 25^th^, 50^th^, and 75^th^ percentiles.

The behavioral variables considered were: level of alcohol consumption, use of illicit drugs and cocaine in the previous six months, use of cell phone applications to find sexual partners in the previous six months (yes or no), STI symptoms in the previous 12 months, receiving STI counseling in the previous 12 months, history of sexual violence and self-reported current risk of HIV infection (none/low or moderate/high). Details about sexual debut were also assessed: the first sexual intercourse (whether it was forced or consensual); the age of first consensual sexual intercourse with a man (under 14 years old, 15–17 years old, or 18 years old or older), and condom use at first consensual sexual intercourse with a man.

Alcohol use was measured using questions formulated by the Alcohol Use Disorders Identification Test (AUDIT)^19^. This test scores questions about the amount and frequency of alcohol use and recent life problems related to alcohol consumption in different ways. Each question scores between 0 and 4 points and scores are combined to give a total score of between 0 and 40. A score of 1 to 7 suggests low-risk consumption, scores from 8 to 14 suggest hazardous or harmful alcohol consumption and a score of 15 or more indicates the likelihood of alcohol dependence (moderate-severe alcohol use disorder). In this study, we categorized alcohol consumption as low-risk consumption or abstaining (0 to 7 points) and risk consumption/probable dependence (8 or more points).

### Statistical analysis

Estimates of the population size of the sample weighting network were performed using the RDS Analyst software version 0.57. Each participant was weighted according to the number of people in their network. In the description of the sample, the unweighted absolute frequencies and the weighted frequencies are presented. Pearson χ^2^ tests were used to assess differences in proportion between the MSM groups.

We performed a weighted univariable and multivariable logistic regression analysis to determine the associations between sociodemographic and behavioral factors and inconsistent condom use in sexual relationships with casual male partners. The magnitude of associations was estimated through the odds ratios, adjusted by the weight of the networks (wOR), with 95%CI. In order to present estimates of association adjusted to each other, all variables were kept in the multivariate model. These analyses were performed using the Statistical Package for the Social Sciences software (SPSS, version 22.0), with the mode of the complex sample for sample weighting.

## RESULTS

Of the total number of respondents, 63.1% reported sexual relationships with casual partners in the six months prior to the survey, 61.1% reported steady partners, and 11.4% commercial partners (data not displayed in the table). The highest percentage of inconsistent condom use was with steady partners (64.5%), followed by casual partners (50.8%) and commercial partners (44.8%) (Table 1). There was a statistically significant difference in condom use between steady partners (p=0.032) and casual partners (p=0.004) when stratified by the age of the men analyzed, indicating that the youngest MSM use lower rates of consistent condom use.

**Table 1:** Type of condom use with different sex partners stratified by age. Data described by unweighted N (weighted %)*.

	Total (n=4,171)	18 to 22 years	23 to 27 years	28 years or more	p-value^†^
Steady partner					
Consistent	893 (35.5)	398 (41.9)	254 (27.6)	241 (30.5)	0.032
Inconsistent	1,780 (64.5)	883 (46.1)	483 (26.7)	414 (27.2)	
Casual partner					
Consistent	1,283 (49.2)	564 (43.5)	372 (27.1)	347 (29.4)	0.004
Inconsistent	1,415 (50.8)	713 (49.1)	358 (23.7)	344 (27.2)	
Commercial partners					
Consistent	330 (55.2)	121 (35.7)	92 (28.2)	117 (36.2)	0.619
Inconsistent	243 (44.8)	95 (37.6)	59 (25.1)	89 (37.3)	

* Weighted percent according to social network size and proportion of men who have sex with men in the city related to total sample; excluding missing data;

𠈂 Pearson’s χ^2^ test.

Among 4,171 participants, 2,722 MSM reported sexual relationships with casual partners in the six months prior to the survey (Table 2). The sample of the study was young (average age of 26 years old, median of 23 years old), high educated (77.5% with 12 years or more of schooling), and non-white (65.3%). Risk consumption or probable dependence on alcohol was verified in 51.3% of the sample, and 51.9% used illicit drugs in the six months prior to the study. More than half of the sample (56.9%) didn't receive any STI counseling in the previous 12 months, and 22.1% presented STI symptoms during this time.

**Table 2:** Sociodemographic and behavioral characteristics of men who reported casual partners in the previous six months. Data described by unweighted N (weighted %).

Characteristics	Total	Consistent	Inconsistent	p-value*
	(n=2,722)	(n=1,292)	(n=1,430)	
Age (years)	26.1 (0.40)	26.8 (0.66)	25.5 (0.46)	<0.001
Socioeconomic class				
A-B	1,329 (47.2)	664 (49.9)	665 (44.7)	0.183
C/D-E	1,393 (52.8)	628 (50.1)	765 (55.3)	
Schooling				
High (12+ years)	2,145 (77.5)	1,053 (81.4)	1,092 (73.7)	0.018
Low (<12 years)	552 (22.5)	230 (18.6)	322 (26.3)	
Skin color				
White	888 (34.7)	431 (36.5)	457 (32.9)	0.341
Non-white	1,827 (65.3)	858 (63.5)	969 (67.1)	
Practicing any religion				
Yes	1,380 (48)	687 (51.6)	693 (44.4)	0.064
No	1,321 (52)	600 (48.4)	721 (55.6)	
Marital status				
Married/in union	231 (8.6)	107 (9.5)	124 (7.8)	0.391
Single/Divorced/Separated	2,483 (91.4)	1,183 (90.5)	1,300 (92.2)	
Alcohol consumption				
Low-risk consumption/abstaining	1,290 (48.7)	638 (51.1)	652 (46.5)	0.242
Risk consumption/probable dependence	1,369 (51.3)	623 (48.9)	746 (53.5)	
Illicit drug use (last 6 months)				
No	1,308 (48.1)	665 (53.3)	643 (43.1)	0.008
Yes	1,396 (51.9)	615 (46.7)	781 (56.9)	
Cocaine use				
No	2,241 (80.3)	1,112 (84.5)	1,129 (76.3)	0.013
Yes	445 (19.7)	162 (15.5)	293 (23.7)	
Use of electronic media to find sexual partners				
No	881 (34.7)	431 (37.9)	450 (31.5)	0.090
Yes	1,822 (65.3)	851 (62.1)	971 (68.5)	
STI symptoms in the last 12 months				
No	2,168 (77.9)	1,089 (81.2)	1,079 (74.6)	0.058
Yes	554 (22.1)	203 (18.8)	351 (25.4)	
Received STI counseling				
Yes	1,094 (43.1)	550 (45.1)	544 (41.1)	0.318
No	1,493 (56.9)	681 (54.9)	812 (58.9)	
History of sexual violence				
No	2,082 (77.8)	1,029 (83.3)	1,053 (72.4)	0.001
Yes	627 (22.2)	262 (16.7)	365 (27.6)	
First sexual intercourse				
Forced	291 (7.3)	121 (5.1)	170 (9.5)	0.003
Consent	2,422 (92.7)	1,166 (94.9)	1,256 (90.5)	
Age at 1^st^ consensual sexual intercourse with a man				
14 years or less	895 (31.5)	384 (28.3)	511 (34.6)	0.022
15–17 years	1,031 (40.0)	472 (38.5)	559 (41.5)	
18 years or more	776 (28.4)	423 (33.2)	353 (23.8)	
Condom use in sexual debut				
No	1,538 (54.4)	625 (41.4)	913 (67.3)	<0.001
Yes	1,142 (45.5)	654 (58.6)	488 (32.7)	
Self-reported current risk of HIV infection				
None/low risk	1,292 (51)	689 (56.4)	603 (45.9)	0.010
Medium/high	1,187 (49)	472 (43.6)	715 (54.1)	

* Proportion homogeneity test based on Pearson’s χ2 statistic or Fischer’s exact test. STI: sexually transmitted infections.

The MSM with inconsistent condom use with casual partners were younger (mean age 25.5 years *vs.* 26.8; p<0.001), and had lower schooling when compared to MSM that used condoms consistently with casual partners (26.3 *vs.* 18.6% with less than 12 years of schooling, respectively, p=0.018). The use of illicit drugs was higher among MSM with inconsistent condom use (56.9 *vs.* 46.7%, p=0.008), as was the use of cocaine (23.7 *vs.* 15.5%, p=0.013). In addition, MSM with inconsistent condom use reported more lifetime sexual abuse episodes (27.6 *vs.* 16.7%; p=0.001) and more of them perceived themselves as at moderate or high risk of acquiring HIV (54.6 *vs.* 43.7%; p=0.012). Differences between the two groups were also verified regarding the first sexual experience. MSM who used condoms inconsistently reported more experience of first forced sexual intercourse (9.5 *vs.* 5.1%, p=0.003), younger age of first consensual sexual intercourse with another man (34.6% initiated with 14 years old or less, *vs*. 28.3%, p=0.022), and lower condom use at first sexual intercourse (67.3% didn't use condom *vs.* 41.4%, p<0.001).

In the weighted univariable regression analysis (Table 3), nine factors were associated with inconsistent condom use: age, schooling, religion, drug and cocaine use, history of sexual abuse during life, first sexual intercourse, age at first consensual sexual intercourse with another man, condom use at sexual debut, and perception of risk for HIV.

**Table 3:** Unadjusted and adjusted estimates associated with inconsistent condom use with casual partners among MSM in Brazil.

	Unadjusted		Adjusted	
	wOR (95%CI)*	p-value	waOR (95%CI)^†^	p-value
Age^‡^	0.97 (0.93–1.00)	0.082	0.94 (0.89–0.99)	0.013
Socioeconomic class				
C/D-E	1.23 (0.91–1.68)	0.183	1.21 (0.85–1.72)	0.290
Schooling				
Low (<12 years)	1.56 (1.08–2.25)	0.019	1.55 (0.99–2.40)	0.051
Race				
Non-white	1.17 (0.85–1.63)	0.341	1.02 (0.70–1.48)	0.928
Practicing any religion				
No	1.34 (0.98–1.82)	0.064	1.17 (0.82–1.66)	0.380
Marital status				
Single/divorced/separated	1.25 (0.75–2.08)	0.392	1.06 (0.55–2.07)	0.857
Alcohol consumption				
Risk consumption	1.18 (0.84–1.66)	0.242	0.92 (0.65–1.31)	0.657
Illicit drug use (last 6 months)				
Yes	1.51 (1.11–2.05)	0.008	1.24 (0.84–1.84)	0.276
Cocaine use (last 6 months)				
Yes	1.70 (1.12–2.59)	0.013	1.06 (0.63–1.80)	0.818
Use of electronic media to find sexual partners				
Yes	1.32 (0.96–1.83)	0.091	1.17 (0.78–1.75)	0.444
STI symptoms in the last 12 months				
Yes	1.47 (0.99–2.19)	0.059	1.19 (0.77–1.84)	0.438
Received STI counseling				
No	1.17 (0.86–1.62)	0.318	1.51 (1.05–2.17)	0.025
History of sexual violence				
Yes	1.90 (1.32–2.75)	0.001	1.49 (0.93–2.39)	0.095
First sexual intercourse				
Forced	1.95 (1.24–3.07)	0.004	0.85 (0.42–1.69)	0.634
Age at sexual debut				
14 years or less	1.71 (1.15–2.54)	0.022	0.98 (0.60–1.60)	0.714
15-17 years	1.51 (1.03–2.20)		1.14 (0.76–1.71)	
Condom use in sexual debut				
Yes	2.91 (2.13–3.99)	<0.001	3.05 (2.12–4.40)	<0.001
Self-reported current risk of HIV infection				
Medium/high	1.52 (1.11–2.10)	0.010	1.51 (1.07–2.14)	0.018

STI: sexually transmitted infections.

* Weighted odds ratio with 95% confidence intervals;

† Weighted adjusted odds ratios with 95% confidence intervals;

‡ Reference value: 26,2. Analysis with an increment of 2 years.

In the multivariable model, five variables were associated with inconsistent condom use: age, schooling, lack of STI counseling, condom use at sexual debut and perception of risk for HIV. Inconsistent condom use was positively associated with low education (wOR: 1.55; 95%CI 0.99–2.40), lack of STI counseling (wOR: 1.51; 95%CI 1.05–2.17), non-use of condoms at sexual debut (wOR: 3.05; 95%CI 2.12–4.40) and moderate and high perceived risk for HIV (wOR: 1.51; 95%CI 1.07–2.14). On the other hand, inconsistent condom use was negatively associated with higher age when the reference value was 26.2 years with an increment of two years (wOR=0.97, 95%CI 0.89–0.99) (Table 3).

## DISCUSSION

In the present study, by estimating condom use in all sexual intercourse in the six months and last intercourse prior to the study, we were able to assess inconsistent condom use with different types of sexual partners. Our results indicate that even in casual relationships, in which condom use tends to be higher compared to those involving steady partners, there is still a significant percentage of MSM who do not use condoms (50.8%) consistently, increasing the level of exposure to HIV and other STIs. This prevalence was higher than in other studies, such as that by Ulrich et al.^20^, conducted in Peru, where 20% of MSM that had more than one partner reported condomless anal intercourse (CLAI) with all of them, or Bavinton et al.^21^, where the prevalence was 30.4% for casual partners in MSM in Bali, Indonesia.

In our study, we demonstrated that younger MSM tend to use less condoms consistently that their older peers, with steady or casual partners. Considering the advances in the treatment and prevention of HIV/Aids, changes are observed in the social representations of the disease. As some studies suggest, and as reported by some key informants in the formative research for this study, the new generations of young homosexuals, born in the post-antiretroviral therapy (ART) era, perceive AIDS as a chronic, but not a fatal disease^22^. This may have implications for how they think about prevention, including consistent condom use.

In our multivariable analyses, low schooling and lower age were associated with inconsistent condom use with casual partners. Sociodemographic characteristics are associated with increased risk behavior for HIV, especially in developing countries. In Brazil, low income and lower education in MSM were associated with higher odds of self-reported HIV positive status in another study^23^. Pascom et al.^24^ found a strong association between younger age, race/color and low education level with not being on treatment or not being virologically suppressed when evaluating the continuum of care for HIV in Brazil. Taken together, these results demonstrate how social determinants play an important role in HIV/AIDS prevention, especially in contexts of high social inequality^2^.

According to our data, a decisive element in adopting condom use is qualified information, particularly that one given in health services or by health professionals. Data on knowledge of STIs in the same MSM population as in the present study indicate that, despite the high level of education in the sample, only 23.7% of men can be classified as having a high level of knowledge about HIV/Aids^22^. However, there are structural barriers in the organization of health services to receive the MSM population. Even in countries where homosexuality is not criminalized, such as Brazil, discrimination and stigma present an important barrier to accessing health services among MSM, especially among the young ones^10^. Due to stigma and discrimination, young MSM experience poorer access to condoms, lubricants, and rapid HIV tests^25^. Also, among those living with HIV, MSM have lower treatment adherence and viral suppression^26^. Non-governmental organizations (NGOs), which have played a fundamental role in disseminating information and prevention since the beginning of the epidemic, especially among key populations, have faced significant financing crises for their actions^27^. In Brazil, unlike what happened between 1990 and 2000, there are few support structures and spaces where young people can exchange information about sexually transmitted diseases. The lack of these spaces was also observed during the formative research of this study. Our data also indicate the importance of sex education before beginning sexual life. This is an important marker of future care, with particular attention to sexual violence against children and adolescents. For this, an organizational structure is needed that has coordinated actions from different sectors, such as education, health, and public safety.

The high prevalence of inconsistent condom use found in these data may be related to changes observed in sexuality in the last years, especially in the case of young MSM. Recently, there has been greater acceptance and visibility of sexual diversity, in addition to greater fluidity in sexual relationships, aided by cell phone applications capable of promoting almost immediate sexual encounters^28^. This set of changes seems to be favoring a more flexible use of condoms. The non-use of condoms may also be related to the phenomenon called “saturation use”, observed mainly in the homosexual population since the beginning of the 1990s, in different parts of the world^29^. There is questioning on the part of the population of MSM to the normative discourse of condom use, materialized through practices such as barebacking (where the risk of HIV infection is deliberately assumed). Also, not using condoms with casual partners may indicate a new way for young MSM to circumvent the stigma that, even after so many years of illness, still links them to HIV/Aids. Studies from the Social Sciences in the 1990s indicated that the gay community was able to reinvent “safe sex” at the beginning of the epidemic^30^, seeking other prevention strategies, such as serosorting, seropositioning, and negotiated security. With the advent of biomedical technologies measures, the same may be happening at the moment. Therefore, it would be important to investigate how inconsistent condom use relates to this and other risk management practices such as PrEP, PEP, and HIV self-testing.

The hypotheses raised in this study are not mutually exclusive, and all of them can contribute to elucidating the observed phenomenon. However, the results presented here have some limitations that must be considered. First, the sample of MSM studied is not representative of the Brazilian population but of the social networks in which the interviewees were recruited. Despite the weighting of the sample, the RDS-type sampling methodology is subject to selection bias, with the sample representing only the networks that the researchers managed to add. However, it is a population that is difficult to access through the usual sampling methodologies. It must be considered that the analyzed data come from a very expressive sample in numerical terms and contemplate an important regional diversity.

Therefore, our findings suggest that, despite being an individual behavior, the use of condoms consistently in MSM with casual partners is related to factors beyond the individual scope. HIV/Aids prevention policies must consider the provision of qualified information for MSM. An important strategy may be the use of combination HIV prevention. Combination prevention programs simultaneously use biomedical, behavioral, and structural interventions to address the needs of a specific population^31^. Evidence suggests that the implementation of combination prevention with PrEP use has been able to reverse the increasing trend of new HIV infections among MSM^32,33,34^. In the Brazilian context, combined prevention, associated with community and civil society mobilization, could positively impact the provision of qualified information about HIV/Aids, and consequently, the use of different prevention strategies by MSM.
